# Comparison between two methods of cleaning and disinfection of digital pulse oximeters in primary care

**DOI:** 10.1590/0034-7167-2024-0485

**Published:** 2026-10-26

**Authors:** Juliana Arruda de Matos, Ivana Bogado Martinez, Érika de Almeida Leite da Silva Teixeira de Souza, Hilda Silva de Souza Barreto, Maria Cristina da Silva Lourenço, Regina Paiva Daumas

**Affiliations:** I Oswaldo Cruz Foundation, National Institute of Infectology Evandro Chagas, Immunization and Health Surveillance Research Unit. Rio de Janeiro, Rio de Janeiro, Brazil; II National Institute of Traumatology and Orthopedics Jamil Haddad. Rio de Janeiro, Rio de Janeiro, Brazil; III Oswaldo Cruz Foundation, National Institute of Infectology Evandro Chagas, Bacteriology and Bioassays Laboratory. Rio de Janeiro, Rio de Janeiro, Brazil; IV Municipal Health Department of Rio de Janeiro. Rio de Janeiro, Rio de Janeiro, Brazil; V Oswaldo Cruz Foundation, National School of Public Health, Germano Sinval Faria Teaching Primary Care Center. Rio de Janeiro, Rio de Janeiro, Brazil

**Keywords:** Disinfection, Primary Health Care, Infection Control, Equipment and Supplies, Detergents., Desinfecção, Atenção Primária à, Saúde, Controle de Infecções, Equipamentos e Provisões, Saneantes., Desinfección, Atención Primaria de Salud, Control de Infecciones, Equipos y Suministros, Saneantes.

## Abstract

**Objectives:**

to compare the efficacy and duration of cleaning and disinfection of digital oximeters with two methods in a primary care setting.

**Methods:**

microbiological culture and adenosine triphosphate assay results were evaluated in oximeters inoculated with *Salmonella typhimurium, Staphylococcus aureus* or *Escherichia coli:* after water and detergent followed by 70% ethanol (method 1); after one-step disinfectant based on benzalkonium chloride and polyhexamethylene biguanide hydrochloride (method 2); and no cleaning and disinfection (control). Effective disinfection was defined as: (1) microbial load reduction of ≥99.99%, (2) negative culture or (3) final microbial load <10^3^.

**Results:**

effective disinfection varied between 33.3% (criterion 2) and 72.2% (criterion 3) and was similar between the two methods, while cleaning and disinfection duration in method 2 was notably shorter.

**Conclusions:**

the efficacy of the two methods was similar and suboptimal, highlighting the challenges associated with the cleaning and disinfection of electronic medical devices in primary care.

## RESUMO

**Objetivos:**

comparar a eficácia e o tempo de execução da limpeza e desinfecção de oxímetros digitais com dois métodos em um ambiente de atenção primária.

**Métodos:**

os resultados da cultura microbiológica e do ensaio de trifosfato de adenosina foram avaliados em oxímetros inoculados com *Salmonella typhimurium, Staphylococcus aureus* ou *Escherichia coli:* após água e detergente, seguidos de etanol a 70% (método 1); após desinfetante de etapa única à base de cloreto de benzalcônio e cloridrato de polihexametileno biguanida (método 2); e sem limpeza e desinfecção (controle). A desinfecção eficaz foi definida como: (1) redução da carga microbiana ≥99,99%, (2)

cultura negativa ou (3) carga microbiana final <10^3^.

**Resultados:**

a desinfecção eficaz variou entre 33,3% (critério 2) e 72,2% (critério 3), sendo semelhante entre os dois métodos, enquanto o tempo de execução da limpeza e desinfecção no método 2 foi notavelmente mais curto.

**Conclusões:**

a eficácia dos dois métodos foi semelhante e inferior ao ideal, destacando os desafios associados à limpeza e à desinfecção de dispositivos médicos eletrônicos na atenção primária.

## RESUMEN

**Objetivos:**

comparar la eficacia y el tiempo de ejecución de la limpieza y desinfección de oxímetros digitales con dos métodos en un entorno de atención primaria.

**Métodos:**

se evaluaron los resultados del cultivo microbiológico y del ensayo de trifosfato de adenosina en oxímetros inoculados con *Salmonella typhimurium, Staphylococcus aureus* o *Escherichia coli:* después de agua y detergente, seguido de etanol al 70 % (método 1); después de un desinfectante de un solo paso a base de cloruro de benzalconio y clorhidrato de poliexametileno biguanida (método 2); y sin limpieza ni desinfección (control). La desinfección eficaz se definió como: (1) reducción de la carga microbiana ≥99,99%, (2) cultivo negativo o (3) carga microbiana final <10^3^.

**Resultados:**

la desinfección efectiva varió entre el 33,3% (criterio 2) y el 72,2% (criterio 3), y fue similar entre los dos métodos, mientras que el tiempo de ejecución de la limpieza y la desinfección en el método 2 fue notablemente más corto.

**Conclusiones:**

la eficacia de ambos métodos fue similar y subóptima, lo que pone de relieve los desafíos asociados a la limpieza y desinfección de dispositivos médicos electrónicos en la atención primaria.

## INTRODUCTION

During the COVID-19 pandemic, home monitoring of peripheral oxygen saturation of patients at increased risk for severe disease was advocated to timely detect hypoxia occurrence^(1)^. The shared use of reusable medical equipment (RME), such as digital oximeters, is one of the transmission mechanisms of microorganisms that cause healthcare associated infections (HAI). Therefore, proper disinfection of RME is crucial to reduce the risk of cross-transmission of pathogens among patients^(2)^.

Sterilization or disinfection are usually two-step processes of which cleaning is a necessary first step. Cleaning removes organic matter, salts, and visible dirt, all of which interfere with microbial inactivation^(3)^. One-step disinfection process is the simultaneous cleaning and disinfection of a fixed surface or non-critical item. A one-step disinfection process may have a better adherence as it is a faster, less complex procedure, easier to train and practice properly^(4)^.

Digital pulse oximeters are non-critical items and require only low-level disinfection before use on another patient, provided they are placed on intact skin and is not soiled with blood or other body fluid^(4)^. Most studies comparing the effectiveness of different methods for low level disinfection are focused on disinfection of environmental surfaces in hospital settings^(5,6)^. It is not clear, however, if this knowledge is applicable to RME. Additionally, given the increasing spread of multidrug resistant (MDR) microorganisms in the community, and the trend for dehospitalization of health services, implementing effective measures for controlling HAI in outpatient facilities, as primary care units, is essential^(7,8)^.

Monitoring the quality of cleaning and disinfection process is an important element for promoting improvements in low-grade disinfection of RME. However, the effectiveness of methods such as microbiological culture, adenosine triphosphate bioluminescence assay (ATP) or fluorescent surface markers has been poorly evaluated for this purpose, and the cut-off values that define non-critical items as clean are not established^(9)^.

## OBJECTIVES

To compare the efficacy of cleaning and disinfecting digital oximeters with water and detergent followed by 70% ethyl alcohol (henceforth “alcohol”) *versus* with a one-step disinfectant based on polyhexamethylene biguanide hydrochloride and benzalkonium chloride (BAC/PHMB); to estimate the correlation of the results of microbiological culture and ATP assay; and to compare the duration of the two disinfection techniques in a primary care setting.

## METHODS

### Design, period and location of the study

This is an experimental, controlled, microbiological challenge study designed to compare the efficacy of two methods for cleaning and disinfection of pulse oximeters. The experiments were performed between January 6th, 2022 and February 3rd, 2022 at the Bacteriology and Bioassays Laboratory of Evandro Chagas National Institute of Infectology of the Oswaldo Cruz Foundation (FIOCRUZ) and at the Central Processing Department of Germano Sinval Faria Teaching Primary Care Center, National School of Public Health, FIOCRUZ, Rio de Janeiro, Brazil.

### Study protocol

We challenged G-Tech Oled Graph digital pulse oximeters (Beijing Choice Electronic Technology Co. Ltd, Beijing, China) with *Salmonella typhimurium* (American Type Culture Collections (ATCC) 14028), *S. aureus* (ATCC 25923) and *Escherichia coli* (ATCC 25922).

We performed three identical experiments with 20 oximeters (n=60). In each experiment, a pair of oximeters was allocated in one of 10 groups, as follows: negative control; *S. typhimurium, S. aureus* and *E. coli* positive controls (PC); *S. typhimurium, S. aureus* and *E. coli* disinfected with alcohol; *S. typhimurium, S. aureus* and *E. coli* disinfected with BAC/PHMB.

Cultures grown for 24 hours on 5% sheep blood Columbia agar (Oxoid, Hampshire, UK) were used. Swabs soaked in a bacterial suspension of approximately 1x10^8^ colony forming units (CFU)/mL of 0.85% saline solution, estimated by DensiCHECK plus (Biomerieux, Craponne, France), was used to inoculate the devices twice, covering the entire internal surface area of the oximeters, including the hinges, with the exception of the oximeters used as a negative control, inoculated only with sterile saline solution. The reapplication of the inoculum occurred after drying the devices.

The oximeters submitted to disinfection with alcohol were initially cleaned with a gauze moistened with water and enzymatic detergent (Ciclozyme Extra, Ciclo Farma Indústria Química, Serrana, Brazil), followed by rinsing with another gauze moistened only with water. After drying with a third gauze, disinfection was performed with a new gauze moistened with 70% ethyl alcohol, by rubbing the external and internal surfaces of the oximeter for 30 seconds. This last step was repeated 3 times, waiting for the equipment to naturally dry.

For the oximeters submitted to disinfection with BAC/PHMB, cleaning and disinfection was performed in a single step by rubbing gauze moistened with the hospital-use disinfectant Surfic^®^ (Profilática Produtos Odonto Médico Hospitalares S.A., Araucária, Brazil), diluted to 0.5%. The active principles of the product are 5.2% alkyl dimethyl benzyl ammonium chloride and 3.5% PHMB.

After drying the inoculum and the disinfection product, when applicable, one half of the internal area of the device was rubbed with a sterile swab for bacterial quantifications, and the other half, with the swab used for the ATP assays. Bacteria were recovered with swabs soaked in 1mL of neutralizing solution (Letheen broth), and homogenized for 30s in vortex. Then, in duplicate, serial 1:10 dilutions were prepared in 96-well plates. Each dilution was seeded on standard Agar for counting (Merck & Co., Inc., Rahway, NJ USA) and incubated for 24h-48h at 37ºC. The number of heterotrophic bacteria in each dilution was estimated from the mean of the CFU of plating in duplicate, and corrections were made to express the result in CFU/device.

The detection of ATP by means of bioluminescence, and expressed in relative light units (RLU), was performed with the Clean TraceTM ATP Surface device (3M Company, Maplewood, USA), according to manufacturer’s instructions.

We cleaned and disinfected 60 devices (30 per technique), in batches of 10 oximeters, to ensure routine processing conditions. We registered the duration of the procedure from the beginning of cleaning and disinfection of the first device until the end of the process of the last device of each batch. After cleaning with soap and water, in the alcohol group, and cleaning/disinfection in the BAC/PHMB group, we performed a visual inspection, and repeated the process in case of visualization of residual dirt.

The devices were handled by researchers wearing gloves during all stages of the study, to avoid cross-contamination with microbiota from their hands.

### Analysis of results and statistics

To assess the effectiveness of the cleaning and disinfection process, we used three different criteria: 1 - estimated reduction of microbial load by 99.99% (10^-4^)^(10)^; 2 - elimination of the bacterial load (vegetative cells); 3 - reduction of the microbial load to less than 10^3(11)^. Reduction in microbial load (criterion 1) was estimated in relation to the positive control.

All data were recorded in Microsoft Excel (Windows 8 version). Data were analyzed using Stata 17.0 (STATA Corp. College Station, Texas, USA). Categorical variables were analyzed using the chi-square test or Fisher’s exact test, when appropriate. Continuous variables were analyzed using the non-parametric Wilcoxon test. The correlation between culture and ATP results was evaluated with the Spearman correlation index. The overall diagnostic performance of the ATP assay was estimated using Receiver Operating Characteristic (ROC) analysis. As a test statistic, p values were calculated. We considered the p value as significant when less than 0.05.

As this work does not involve collection of human data, ethical revision and approval and patient consent was not applicable.

## RESULTS

There was a statistically significant difference in culture and ATP assay results between oximeters submitted to cleaning and disinfection and positive controls; there was no difference between the two cleaning and disinfection methods (Tables 1 and 2).

**Table 1:** Median (interquartile range) of culture results, expressed in colony forming units/ml, for each group of experiments - non-contaminated oximeters (negative control), oximeters inoculated with *Escherichia coli*, *Staphylococcus aureus* and *Salmonella typhimurium* not subjected to disinfection (positive control) and after disinfection with 70% ethyl alcohol or benzalkonium chloride and polyhexamethylene biguanide hydrochloride

	Negative Control (n=6)	Positive Control (n=17)	Alcohol (n=18)	BAC/PHMB (n=18)	*p* value^**^		
					Alcohol vs. positive control	BAC/PHMB vs. positive control	Alcohol vs. BAC/PHMB
*E. coli*	0 (0-0)	12500 (11750-18000)	0 (0-0)	12.5 (0-25)	0.004	0.004	0.424
*S. aureus*		575000 (232500-2100000)	1625 (825-6750)	687.5 (50-1750)	0.002	0.011	0.331
*S. typhimurium*		33250^*^ (26750-67500)	75 (0-625)	75 (50-750)	0.004	0.004	0.567

BAC/PHMB - benzalkonium chloride and polyhexamethylene biguanide hydrochloride;

* 5 positive control experiments of S. typhimurium were carried out - the 6th experiment was discarded;

** Wilcoxon test.

**Table 2:** Median (interquartile range) of adenosine triphosphate assay results, expressed in relative light units, for each group of experiments-non-contaminated oximeters (negative control), oximeters inoculated with *Escherichia coli, Staphylococcus aureus* and *Salmonella typhimurium* not subjected to disinfection (positive control) and after disinfection with 70% ethyl alcohol or benzalkonium chloride and polyhexamethylene biguanide hydrochloride

Microorganism	Negative Control (n=6)	Positive Control (n=18)	Alcohol (n=18)	BAC/PHMB (n=18)	*p* value^*^		
					Alcohol vs. positive control	BAC/PHMB vs. positive control	Alcohol vs. BAC/PHMB
*E. coli*	73.5 (21-109)	1819 (1722-1988)	55.5 (49-72)	36.5 (33-47)	0.002	0.002	0.065
*S. aureus*		3021.5 (1059-3354)	62 (31-79)	51.5 (31-68)	0.002	0.002	0.974
*S. typhimurium*		1927 (568-3229)	46.5 (33-95)	47.5 (45-52)	0.002	0.002	0.900

BAC/PHMB - benzalkonium chloride and polyhexamethylene biguanide hydrochloride;

* Wilcoxon test.

Culture and ATP assay results showed a positive and significant correlation, with a Spearman correlation index of 0.648 (p<0.001).

The analysis of the efficacy of cleaning and disinfection according to the three different criteria is shown in Table 3. There was a statistically significant difference in the percentage of effective disinfection according to the microorganism, being lower in oximeters contaminated with *S. aureus* (criteria 2 and 3) and higher in those contaminated with *E. coli* (criteria 1 and 2), regardless of the disinfection method used. There was no difference in the percentage of effective disinfection between the two methods (alcohol vs BAC/PHMB). Moreover, ATP assay results from oximeters classified as effectively disinfected were not significantly different from those with ineffective disinfection, regardless of the criterion used. There was a low discriminatory power of the ATP assay to define the appropriateness of disinfection, regardless of the criterion used, although there was a slightly better performance with criterion 3, the least strict one (area under receiver operating curve (ROC) (CI95%) = 0.57 (0.37-0.77), 0.52 (0.32-0.73) and 0.67 (0.47-0.87) for criterium 1, 2 and 3, respectively (Figure 1)). It was not possible to determine an optimized ATP assay cutoff point to define effective cleaning and disinfection according criteria 1 and 2; the best cutoff according to criterion 3 was 47 RLU, with sensitivity of 80.0% and specificity of 53.9%.

**Table 3:** Comparative analysis between oximeters whose disinfection was considered effective or not according to three different criteria

Variable	Criterion 1 - estimated microbial load reduction of >99.99%			Criterion 2 - negative culture			Criterion 3 - reduction of microbial load to less than 10^3^		
	Effective disinfection n=13 (36.1%)	Ineffective disinfection n=23 (63.9%)	*p* value	Effective disinfection n=12 (33.3%)	Ineffective disinfection n=24 (66.7%)	*p* value	Effective disinfection n=26 (72.2%)	Ineffective disinfection n=10 (27.8%)	*p* value
Microorganism by desinfection method - n (%)									
*E. coli*	8 (66.7%)	4 (33.3%)	**0.011**	8 (66.7%)	4 (33.3%)	**0.007**	11 (91.7%)	1 (8.3%)	0.115
Alcohol	5 (83.3%)	1 (16.7%)	0.545	5 (83.3%)	1 (16.7%)	0.545	6 (100.0%)	0 (0.0%)	1.000
BAC/PHMB	3 (50.0%)	3 (50.0%)		3 (50.0%)	3 (50.0%)		5 (83.3%)	1 (16.7%)	
*S. aureus*	2 (16.7%)	10 (83.3%)	0.143	1 (8.3%)	11 (91.7%)	**0.031**	5 (41.7%)	7 (58.3%)	**0.007**
Alcohol	0 (0.0%)	6 (100.0%)	0.455	0 (0.0%)	6 (100.0%)	1.000	2 (33.3%)	4 (66.7%)	1.000
BAC/PHMB	2 (33.3%)	4 (66.7%)		1 (16.7%)	5 (83.3%)		3 (50.0%)	3 (50.0%)	
*S. typhimurium*	3 (25.0%)	9 (75.0%)	0.468	3 (25.0%)	9 (75.0%)	0.709	10 (83.3%)	2 (16.7%)	0.438
Alcohol	3 (50.0%)	3 (50.0%)	0.182	3 (50.0%)	3 (50.0%)	0.182	5 (83.3%)	1 (16.7%)	1.000
BAC/PHMB	0 (0.0%)	6 (100.0%)		0 (0.0%)	6 (100.0%)		5 (83.3%)	1 (16.7%)	
Disinfection method (overall) - n (%)									
Alcohol	8 (44.4%)	10 (55.6%)	0.489	8 (44.4%)	10 (55.6%)	0.289	13 (72.2%)	5 (27.8%)	0.644
BAC/PHMB	5 (27.8%)	13 (72.2%)		4 (22.2%)	14 (77.8%)		13 (72.2%)	5 (27.8%)	
ATP analysis in RLU - median (IQR)	49 (32-61)	49. (34-77)	0.499^*^	49.5 (35.5-66.5)	48 (33.5-72.5)	0.823^*^	45.5 (33-68)	60.5 (47-91)	0.128^*^

BAC/PHMB - benzalkonium chloride and polyhexamethylene biguanide hydrochloride; ATP - adenosine triphosphate bioluminescence assay; RLU - relative light units; IQR - interquartile range;

* Wilcoxon test; otherwise, Fisher exact test.

**Figure 1:**
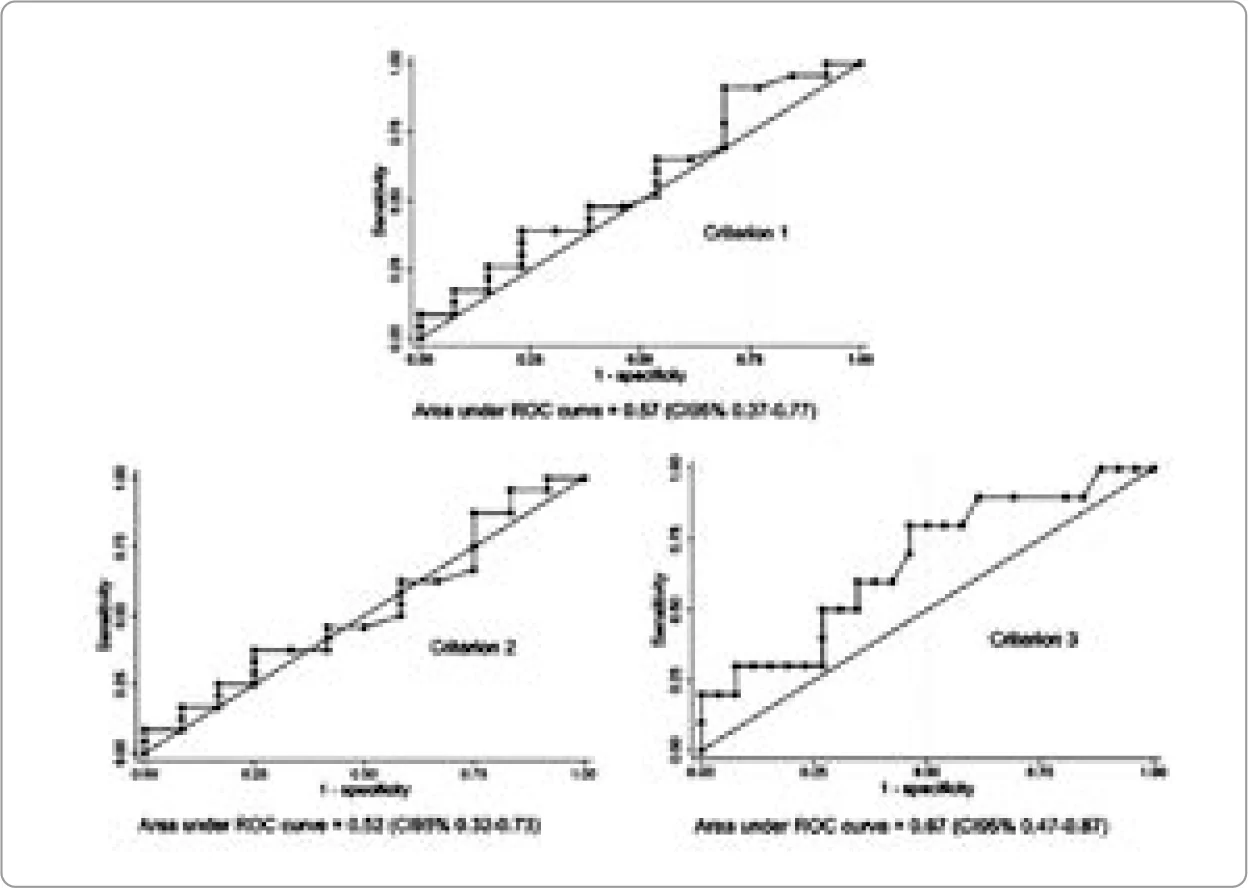
Receiver operating curve (ROC) analysis of the performance of the adenosine triphosphate bioluminescence assay (ATP) in identifying effective cleaning and disinfection of digital oximeters according to three criteria

The mean time required for disinfection of each batch of 10 oximeters with alcohol (including the time for cleaning with detergent and water) was almost twice (ratio = 1,97) that for disinfection in one-step with BAC/PHMB (23, 24 and 28 minutes vs. 11, 12 and 15 minutes, respectively). Thus, the estimated average time to disinfect a single device was 2.5 minutes for alcohol versus 1.26 minutes for BAC/PHMB.

## DISCUSSION

Our study showed a low efficacy of the two processes of cleaning and disinfection of digital oximeters evaluated, regardless of the effective cleaning criterion adopted, with no significant difference between both.

The processing of RME has become increasingly complex as the design of these products becomes increasingly sophisticated^(10)^. Among the challenges are electronic equipment, which are not suitable to chemical disinfection by immersion, in addition to being composed of different materials and having recesses (e.g. hinges), what make cleaning and disinfection of RME much more difficult than environmental surfaces.

Nandy et al.^(12)^ evaluated the effectiveness of cleaning and disinfection of oximeter sensors, artificially contaminated with 1x10^8^ CFU/ml of vegetative bacteria with or without sebum, using different products. Wiping with 70% isopropanol, the manufacturer’s recommended disinfection method, removed approximately only 90% of vegetative bacteria in the absence of sebum. Wipes impregnated with hypochlorite showed the best results, and the commercial wipe based on BAC 0.28%, the worst, but the exact percentage of inoculum removal was not clarified. The authors conclude that there is a need to define methods for effective disinfection of reusable sensors^(12)^.

Most registered disinfectants have a 10-minute label claim. However, multiple investigators have demonstrated the effectiveness of these disinfectants against vegetative bacteria, yeasts, mycobacteria and viruses at exposure times of 30-60 seconds^(4)^. The variability in the time of exposure to the products applied through wiping, however, makes it difficult to standardize this technique, and may be responsible, at least in part, for the variability of results found in the literature.

In Boom et al.^(13)^, ampoules and multidose vials were subjected to different alcohol disinfection techniques. The addition of an immersion step significantly increased the efficacy of the rubbing technique with two wipes^(13)^. The authors recommend the use of commercially available alcohol impregnated sterile wipes, to eliminate the effect of insufficient wetting of sterile gauzes with alcohol^(13)^.

Interestingly, Tyski et al.^(14)^ used an environmental cleaning model to evaluate the effectiveness of 12 different disinfection wipes, and showed greater effectiveness in the disinfection process with the self-made wipes, when compared to the commercial ones. Wipes impregnated with alcohol showed lower efficacy than those impregnated with quaternary ammonium. In this study, where the surface was challenged with 10^9^ CFU/mL of vegetative bacteria in the presence of a bovine albumin solution and sheep erythrocytes, the least effective products (log10 reduction of less than 5) were commercial wipes containing ethanol and sodium hypochlorite^(14)^.

A Brazilian group evaluated the performance of BAC/PHMB 5.2% /3.5% diluted to 1% for disinfection of environmental surfaces in an intensive care unit (ICU), and demonstrated persistence of MDR pathogens in different areas of the studied ICU, even after disinfection with the BAC/PHMB-based product^(15)^.

Other remarkable finding of our study was the greater efficacy of both disinfection methods against GNB, especially *E. coli*, compared to *S. aureus*, a finding that has been corroborated by other studies.

*Serratia marcescens, E. coli* and *Salmonella typhosa* were killed in 10 seconds by all concentrations of ethanol from 40% to 100%, while *S. aureus* and *Streptococcus pyogenes* required concentrations between 60%-95% for being killed after the same exposition time, highlighting the greater resistance of gram positive cocci to ethanol^(4)^. In the study by Nandy et al.^(12)^, disinfection efficiency was worse for oximeter sensors contaminated with *S. aureus*, compared to *Burkholderia thailandensis* and *Yersinia pseudotuberculosis*. Among the possible explanations for the increased adhesion of *S. aureus*, the authors mentioned a more tightly adhesion of staphylococci to silicone than to acrylic or other biomaterials, most likely owing to silicone’s higher hydrophobicity and roughness. The internal surfaces of the digital pulse oximeter used in the present study are covered by medical silicone, which may help explain the greater resistance of *S. aureus* to disinfection.

Another interesting finding was that cleaning and disinfection with a one-step disinfectant were faster than cleaning with soap and water followed by disinfection with 70% alcohol, with the same efficacy, which makes it an attractive alternative for disinfection of non-critical items in primary care.

ATP is the basic energy source for all living cells. Consequently, the presence of ATP on surfaces is an indicator of organic matter, including, but not limited to, microbial contamination. The utility of ATP bioluminescence assays to measure effectiveness of cleaning after environmental surfaces disinfection in hospital settings have been extensively studied, with conflicting results^(16)^. However, research about monitoring of cleaning and disinfection of RME utilizing ATP technology is limited^(17)^.

Reese et al.^(17)^ carried out a quality improvement project on RME cleanliness using educational tools and ATP assay monitoring and feedback to healthcare staff on a regular basis. The median RLU decreased 75% (860 vs. 218; *p*< 0.001) in six months, and an additional 19% after one year, suggesting a correlation between ATP assay and cleanliness, even though the ATP assay results were not compared against culture results. An interesting finding of this study is that, while most of the equipment demonstrated reduction in overall RLU values over time, this was not the case with pulse oximeters. The authors hypothesized that the small area on the pulse oximeter made it difficult to adequately clean, resulting in a higher RLU^(17)^.

ATP bioluminescence assays have some limitations such as impossibility to identify the organism and no direct relation between RLU and bacterial load^(18)^. In the present study, although there was a significant correlation between the number of colonies and the amount of ATP detected, the performance of the ATP assay in identifying effective cleaning and disinfection was low.

Furlan et al.^(6)^ compared three methods to evaluate the adequacy of cleaning and disinfection of environmental surfaces, with a one-step disinfectant, in an outpatient clinic in Brazil: visual inspection, microbiological culture for determination of aerobic colony count (ACC) and ATP assay. Surfaces were considered clean when ACC results were <2.5 CFU/cm2. There was a poor performance of ATP assay: a positive and significant correlation between ACC and ATP assay was achieved only for 2 out of 5 surfaces evaluated. The best cutoff point of ATP assay, determined by ROC analysis, was 49 RLU, with low sensibility and specificity (53.3% and 66.7%, respectively). These findings are highly comparable to ours.

A systematic review of effectiveness of ATP bioluminescence to assess hospital cleaning, when compared to microbiological methods, included 14 studies, 11 of which identified a significant correlation between the two methods, although poor or not complete for five. Sensitivity and specificity of ATP assay were not reported. The authors concluded that ATP bioluminescence is not a standardized methodology, since each tool has different benchmark values, not always clearly defined^(16)^. The criterion used to define environmental surfaces as clean in all studies included in this systematic review, and in the literature in general, was a result of less than 2.5 CFU/cm2 with the ACC method^(19)^. However, this method is applicable only to large, plain environmental surfaces, since it consists of applying contact plates to the testing surface, which is not feasible to most RME. Such a microbiological benchmark for defining a non-critical item as clean after low-level disinfection has not been defined, which hampers comparisons between studies and the interpretation of results of ATP assay and other methods for disinfection monitoring.

### Study limitations

Our study has some limitations. We did not include organic matter, like sebum or sweat, to simulate contamination after use of digital oximeters. Another limitation was not having included a group submitted to wiping with water, to take into account the effect of friction itself. In both cases, the efficacy of the disinfecting methods could have been overestimated.

### Contributions to the field of Nursing

Our study had the strength of having used different criteria for considering a non-critical item as clean, counterbalancing the uncertainty regarding the best microbiological benchmark of effective low-grade disinfection of RME. In addition, we used clinically relevant pathogens as contaminating bacteria, increasing the extrinsic validity of the study. Our study adds information about cleaning and disinfection of a shared and reusable electronic medical device in a primary care setting, where infection control practices are poorly studied.

## CONCLUSIONS

The duration of cleaning and disinfection with BAC/PHMB was shorter than that of water and detergent followed by 70% ethanol, but the efficacy of the two methods was similar and suboptimal. These findings highlight the challenges associated with the disinfection of electronic medical devices in primary care and draw attention to the importance of infection control in this context as well.

## Data Availability

The research data are available in a repository: https://doi.org/10.48331/scielodata.G7FPZG.
